# Sucrose Exposure in Early Life Alters Adult Motivation and Weight Gain

**DOI:** 10.1371/journal.pone.0003221

**Published:** 2008-09-17

**Authors:** Cristianne R. M. Frazier, Peggy Mason, Xiaoxi Zhuang, Jeff A. Beeler

**Affiliations:** 1 Committee on Neurobiology, University of Chicago, Chicago, Illinois, United States of America; 2 Department of Neurobiology, University of Chicago, Chicago, Illinois, United States of America; Temasek Life Sciences Laboratory, Singapore

## Abstract

The cause of the current increase in obesity in westernized nations is poorly understood but is frequently attributed to a ‘thrifty genotype,’ an evolutionary predisposition to store calories in times of plenty to protect against future scarcity. In modern, industrialized environments that provide a ready, uninterrupted supply of energy-rich foods at low cost, this genetic predisposition is hypothesized to lead to obesity. Children are also exposed to this ‘obesogenic’ environment; however, whether such early dietary experience has developmental effects and contributes to adult vulnerability to obesity is unknown. Using mice, we tested the hypothesis that dietary experience during childhood and adolescence affects adult obesity risk. We gave mice unlimited or no access to sucrose for a short period post-weaning and measured sucrose-seeking, food consumption, and weight gain in adulthood. Unlimited access to sucrose early in life reduced sucrose-seeking when work was required to obtain it. When high-sugar/high-fat dietary options were made freely-available, however, the sucrose-exposed mice gained more weight than mice without early sucrose exposure. These results suggest that early, unlimited exposure to sucrose reduces motivation to acquire sucrose but promotes weight gain in adulthood when the cost of acquiring palatable, energy dense foods is low. This study demonstrates that early post-weaning experience can modify the expression of a ‘thrifty genotype’ and alter an adult animal's response to its environment, a finding consistent with evidence of pre- and peri-natal programming of adult obesity risk by maternal nutritional status. Our findings suggest the window for developmental effects of diet may extend into childhood, an observation with potentially important implications for both research and public policy in addressing the rising incidence of obesity.

## Introduction

Almost two-thirds of American adults are either overweight or obese and the incidence of childhood obesity is rising rapidly [1]–[4]. The cause of the recent and dramatic rise in obesity in western societies is not fully understood, but its appearance in a genetically stable population indicates that environmental factors play a critical role [5]–[7]. In western societies, the increased availability of tasty, energy-rich foods together with a reduced requirement for energy expenditure is believed to promote a net positive energy balance resulting in increased body mass and obesity [5], [8]. The availability of these foods, however, does not entirely account for their widespread (over-)consumption. Palatable, high-calorie foods are widely available because there is a demand for them, though marketing practices undoubtedly contribute to this, especially among children [9]–[11]. Moreover, the widespread availability of high fat and high sugar foods does not mean that healthier alternatives are unavailable, though this may vary by region and socioeconomic status [12]–[18]. Human beings, though influenced by environmental factors [7], respond to their environment with motivated, goal-directed behavior [19]–[21], often seeking out foods they know to be poor choices, sometimes even when they resolve to eat better or less. These adult behaviors are often resistant to change [22]. Understanding the factors that shape adult food-related motivation and its contribution to weight gain would greatly aid in the development of preventative measures in tackling the problem of obesity.

Efforts to understand the recent rise in obesity focus on discerning the relative contributions of, or interactions between, genetic and environmental factors in adulthood [6], [7], [19], [23], [24]. Developmental contributions and interactions are only recently being investigated and have focused almost exclusively on maternal over- and under-nutrition affecting the fetal or neonatal nutritional environment. Arising from the fetal origins hypothesis, termed the ‘thrifty phenotype,’ first proposed by Hales, Barker and colleagues [25], [26], this work proposes that pre- and peri-natal nutritional status developmentally programs the organism's adult metabolism and energy balance to form a “predictive adaptive response” [27]. This work has clearly demonstrated that early nutritional status can alter later energy balance behavior and body weight [28]–[36]. The window during which such programming occurs, however, is not well defined. Whether a developmental window remains open during childhood and adolescence allowing nutritional experience during this time to substantively shape subsequent adult energy balance remains an open question. As children's diets in contemporary western society tend to be replete with high fat and sugar foods, such developmental effects could potentially compound the obesity epidemic or, alternatively, offer a potential approach to addressing it.

Human studies examining childhood diet and obesity are equivocal, providing some evidence that childhood diet can contribute to obesity risk [37] but also showing that restricting children's diets increases obesity risk [38], though these studies generally do not examine adult outcome. Given the difficulty inherent in human studies, including the challenge of maintaining adequate experimental control and the expense and delay inherent in human longitudinal studies, an animal model would greatly facilitate investigations into the relationship between early dietary experience and adult obesity.

Like many humans, C57BL/6 mice show a strong preference for sugar and fat and become obese and develop diabetes when given chronic access to a high-sugar/high-fat diet [39]–[42]. The C57BL/6 mouse shares with humans the ‘thrifty genotype,’ a putative genetic predisposition to store calories whenever food is readily available [8], [40]. Here we used C57Bl/6 mice to directly test the effect of early post-weaning experience on adult motivated behavior and obesity risk. We focused on sucrose exposure in early life since sucrose is a potent natural reinforcer [43] and because the complex mechanisms that have evolved to regulate sugar metabolism interact with motivational and reward systems [44]. We hypothesized that early experience with sucrose can alter adult motivated behavior and thereby may constitute an important factor determining adult feeding behavior and energy balance.

We show that unlimited access to sucrose early in life reduces motivation to acquire sucrose, but only when work is required to obtain it. When high-sugar/high-fat foods were made freely-available, mice exposed to sucrose early in life preferred and consumed this food as much as non-exposed animals, but in this environment gained more weight than controls. These data provide clear empirical support for the often asserted but rarely demonstrated link between childhood diet and later adult feeding behavior and body weight and suggest that the impact of early diet on adult obesity risk may be contingent upon the adult environment. These findings additionally suggest that the window for developmental programming in response to nutritional environment extends beyond gestation and suckling.

## Materials and Methods

### Animals

Ten breeding pairs of C57BL/6 mice (Jackson Laboratories, Ben Harbor) were set up and each litter was weaned at 21 days and distributed evenly between the experimental early environmental conditions (unlimited sucrose exposure and no sucrose exposure). Animals were housed under standard conditions throughout. The sucrose exposure group, upon weaning, had 20 mg sucrose pellets (Bio-Serv, Frenchtown, NJ) continuously and freely available within their homecage. There were two cohorts born 3 weeks apart. The sucrose was removed from all sucrose exposure mice at the same time, resulting in two cohorts with either 4 or 7 weeks exposure to sucrose. They were maintained on standard *ad libitum* chow thereafter, except during the dietary challenge in which they were offered a high sugar/high fat option in addition to standard chow. Both males and females were included and distributed approximately equally.

### Behavior Tests

All experiments were carried out during the light period (06:00–18:00). When food restriction was used, mice were given 2 hours per day access to food immediately following the behavior test. Water was available *ad libitum*.

#### Open field

Each mouse was placed in an acrylic open field chamber 40 cm long×40 cm wide×37 cm high (Med Associates, St. Albans, VT). Illumination was 21 lux. Infrared beams recorded the animals' locations and paths (locomotor activity) as well as the number of rearing movements (vertical activity). Data were collected in 5 minute bins during 60 min trials. The chambers were cleaned with 70% ethanol between all trials.

#### Wheel running

Mice were singly housed each with a 4.5″ wire mesh wheel (Mini Run-a-Round, Pets International, Ltd., Elk Grove Village, IL). Two counter-balanced magnets (Digi-key, Thief River Falls, MN) were placed on 3/8 inch stainless steel strips attached to the wheel (McMaster Carr Supply Co, Chicago, IL). The wheel was situated in the cage such that a magnetic switch closes (Digi-key) at every pass of a magnet. Data were collected using Vitalview acquisition software, QA-4 activity input modules, and DP-24 data ports, (Mini-mitter Co., Sun River, OR). Data were collected in minute bins throughout the one week experiment.

#### Operant tasks

The progressive ratio (PR) operant task and the concurrent choice task were conducted in operant conditioning chambers (Med Associates, St Albans, VT), 5 days per week in 90 min sessions for the progressive ratio task and 30 min sessions for the concurrent choice task. In the PR experiment, mice were first trained under a fixed ratio one schedule (FR1, every press rewarded) with only the active lever extended. During the first two days of training, food pellets (20-mg sucrose pellets, Bio-Serv, Frenchtown, NJ) were also delivered into the food receptacle on a random interval 60 s schedule with intervals ranging between 0 and 120 seconds. When mice reached a criterion of 30 lever presses in less than 45 min on two consecutives days they were shifted to a PR3 schedule for two days before PR7 testing began. In progressive ratio, the number of lever presses required to earn a pellet is incremented by 3 (PR3) or 7 (PR7) after each reward so that each subsequent pellet becomes more costly. Mice were tested under both food-restricted and non-food-restricted conditions. Three parameters were recorded: the breakpoint, defined as the last ratio completed, and number of lever presses on the active and inactive levers. The concurrent choice task was adapted from the protocol used by Cousins and Salamone [45] in rats. Tests were conducted under food restriction. Mice had the choice between lever pressing for a more preferred food (FR35, every 35 lever presses delivers a 20 mg sucrose pellet) or consuming a less preferred standard rodent chow that was concurrently and freely available on the floor of the operant box. This experimental arrangement (“choice condition”) was used on day 1, 3 and 5 of each week; and on days 2 and 4, only the FR35 was available (“no choice condition”). Testing lasted 1 week. The number of lever presses, the total amount of food consumed (pellet and lab chow in g) and the percentage of food obtained by lever pressing were calculated.

#### Sucrose preference and extinction

Mice were singly housed in cages that included two identical water bottles (15 ml round bottom polypropylene tubes with rubber stopper on the mouth and a sipper tube with ball-bearing), one of which contained sucrose solution at varying concentrations in the course of the experiment (0%, 0.2%, 5%, 10% and 15%). Each concentration of sucrose was provided for four days. The positions of the bottles were rotated daily to counter-balance potential position preferences. The bottles were weighed daily and the amount consumed from each recorded. A cage without mice was maintained and weighed daily to track spillage, which remained minimal and is not reported. Preference as a percent was calculated by dividing the amount consumed from the sucrose bottle from the total amount consumed from both bottles. After the final 15% concentration, all bottles were washed thoroughly and water placed in all bottles and the test was continued for an additional two days to determine the rate of extinction of the preference for the previously sucrose filled bottle.

### Glucose Challenge

Mice were fasted for 21 hours. Tail bleeds were used to sample blood glucose and measured using the Accu-Check Active Meter (Roche). After a baseline fasting glucose level was obtained, mice were challenged with 2 g/kg dextrose prepared in 0.9% saline and blood glucose checked at 30, 65 and 90 minutes.

### High-Sugar/High-Fat Dietary Challenge

Mice were singly housed and provided both standard chow and equal amounts of various Nestlé® chips (butterscotch, milk chocolate, white chocolate, peanut butter) *ad libitum*. The mice were weighed and consumption measured weekly during the three week dietary challenge. Mice had *ad libitum* access to water. Subsequent to the challenge, mice were returned to the standard chow diet and group housing. Metabolic efficiency was calculated by dividing the total weight gained during the challenge by the kcal consumed to yield weight gain per kcal. Caloric content of chow and Nestlé® chips were obtained from the manufacturers' websites.

All animal procedures were approved by the Institutional Animal Care and Use Committee at The University of Chicago.

## Results

**Figure 1:**
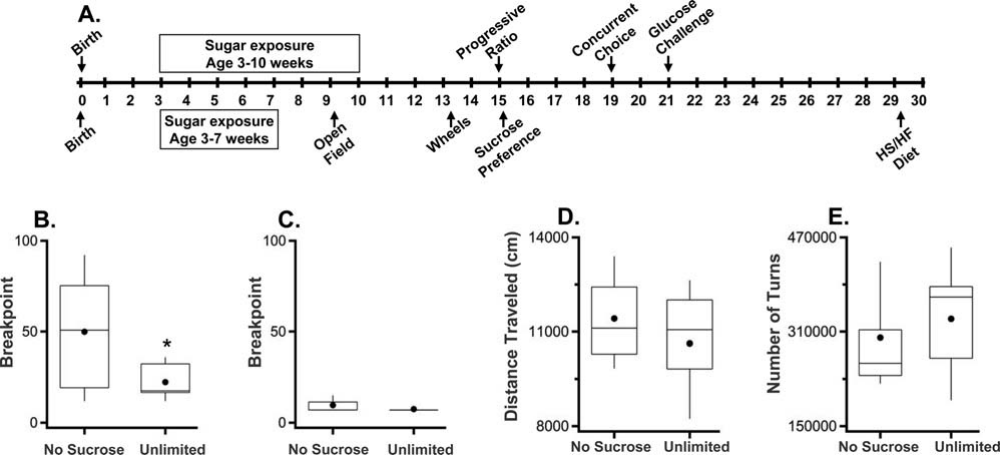
Experimental timeline, progressive ratio and activity. (a) Timeline indicating the order of experimental manipulations and tests. Two cohorts of mice were used and administered the experimental manipulation and testing procedures as indicated above and below the timeline. Breakpoint in progressive ratio (PR7) for the sucrose exposed and non-sucrose exposed mice tested under (b) food restriction and (c) sated conditions (n = 8). (d) Total distance traveled in the open field (1 hr) (n = 7). (e) Total number of wheel turns in 1 week (n = 7, 8). Box plots: Middle lines represent the median values, the top and bottom of the boxes represent the 25th and 75th percentiles, the whiskers represent the 10th and 90th percentiles and the dots represent mean values ; * p = .05.

Thirty male and female C57BL/6 mice were weaned onto standard chow and half of the animals were given concurrent exposure to unlimited amounts of sucrose (20 mg pellets) in their homecage for four weeks (n = 7) or seven weeks (n = 8; Figure 1a). This post-weaning period of development approximately corresponds to childhood through adolescence or early adulthood. Following this manipulation, there was no difference in weights between the two groups (4 wk group, F_(1,9)_ = 2.96, p = 0.1; 7 wk group, F_(1,12)_ = 0.35, p = 0.6) as expected since sucrose consumption alone does not generally cause weight gain in rodents [40], [46], [47] or humans [48]–[50]. Thereafter, all animals were maintained under standard conditions with *ad libitum* access to standard chow. The groups were tested as indicated in Figure 1a.

### Behavioral effects of early sucrose exposure

Five weeks after the termination of sucrose exposure, adult animals were tested for their willingness to work to acquire sucrose rewards in the progressive ratio test. In this instrumental task, the number of active lever presses required to obtain a sucrose reward is increased incrementally after each sucrose pellet earned. The maximal number of presses for which the animal earned a reward is termed the breakpoint and reflects an animal's willingness to work for a reinforcer. Mice given unlimited access to sucrose early in life exhibited a lower breakpoint than animals not exposed to sucrose (Figure 1b; F_(1,14)_ = 4.61, p = 0.05). This effect was only observed if mice were food-restricted (Figure 1c; sated condition: F_(1,14)_ = 2.11, p = 0.2). Interestingly, though not statistically significant, the unlimited sucrose exposure group tended to press less on the inactive lever as well, which does not yield reward, than did the no sucrose exposure group (food- restricted condition, F_(1,14)_ = 3.38, p = 0.09, data not shown). Inactive lever-pressing can be interpreted as either an indicator of general activity or as an expression of the animals' exploratory strategy; that is, how often they check the ‘other lever’ to see if reward contingencies have changed. Baseline locomotor activity measured in the open field test (Figure 1d; F_(1,12)_ = 0.52, p = 0.5) or in homecage wheel-running (Figure 1e; F_(1,13)_ = 0.28, p = 0.6) was not affected by sucrose exposure, suggesting no difference between the two groups in basal locomotion and goal-directed energy-expenditure (wheel running), although these activity measures were not taken under food restriction. Taken together, these behavior results indicate that mice given unlimited access to sucrose in early life exhibit reduced sucrose-seeking behavior in adulthood as indicated by a lower breakpoint.

**Figure 2:**
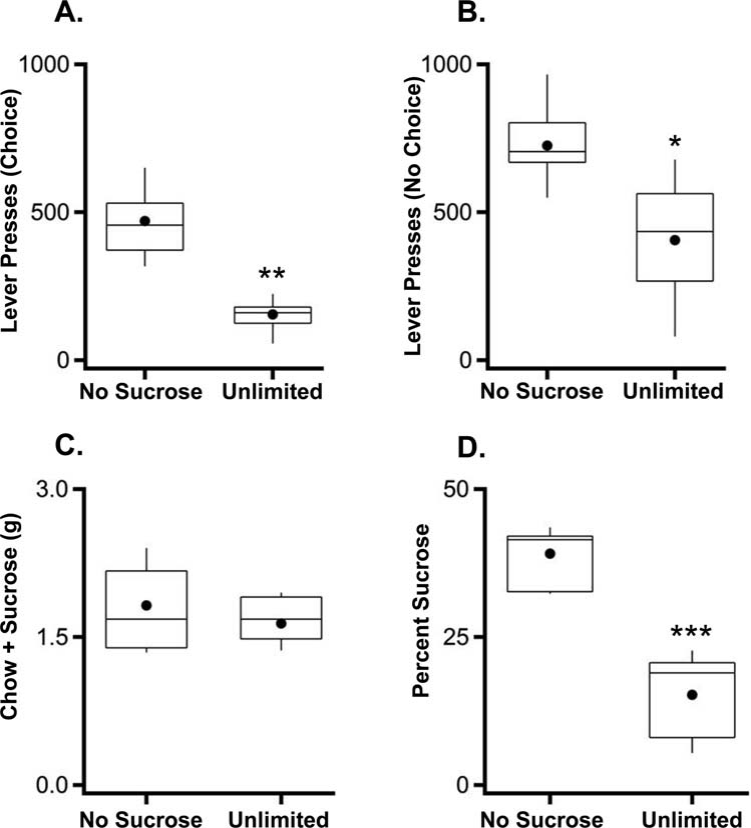
Concurrent choice. Average lever presses during (a) choice and (b) no choice conditions (n = 8). (c) Total food consumed (earned sucrose+freely available chow) during choice sessions (n = 8). (d) Percentage of total intake comprised of earned sucrose pellets during choice sessions (n = 8). Box plots: as described in figure 1; * p<.05; **p<.005; *** p<.0001.

Given that unlimited sucrose exposure early in life reduced sucrose-seeking in adulthood, we asked how the mice would behave given a choice between freely available, standard chow and sucrose that had to be earned. In this concurrent choice paradigm, mice were tested in two conditions termed ‘choice’ and ‘no choice’ on alternating days. In the choice condition, mice could either work (lever-press) for sucrose pellets or eat standard chow freely available. In the no-choice condition, there was no freely available chow. There was no difference in total food consumption (chow plus sucrose) between the groups in the choice condition (Figure 2c; F_(1,14)_ = 0.59, p = 0.5). Consistent with the results from the food-restricted progressive ratio test, mice exposed to unlimited sucrose in early life worked less to obtain sucrose in both the choice and no choice conditions (Choice Condition, Figure 2a; F_(1,14)_ = 26.25, p<0.001; No Choice Condition, Figure 2b; F_(1,14)_ = 5.67, p = 0.03) and showed enhanced preference for freely-available standard chow in the choice condition (Figure 2d; F_(1,14)_ = 38.07, p<0.0001). Thus, unlimited access to sucrose early in life reduced motivation for sucrose rewards and preferentially enhanced consumption of freely-available food over more palatable food that required work to obtain.

**Figure 3:**
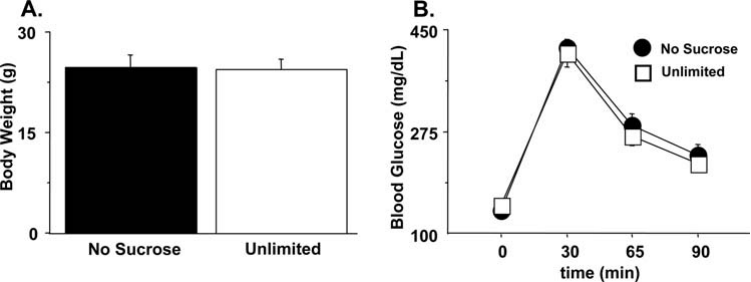
Glucose challenge. (a) Weights after a 21 hour fast and prior to glucose challenge. (b) blood glucose levels at fasting baseline (timepoint 0) and following i.p. injection of dextrose (2 g/kg). N = 6 (no sucrose) and 8 (unlimited).

Following the concurrent choice test, we evaluated glucose metabolism in this cohort of mice. After 21 hours of fasting, there were no differences in weight between the groups (Figure 3a; F_(1,14)_ = .011, p = 0.917) and no difference in either fasting glucose levels (Figure 3b; baseline, F_(1,12)_ = .552, p = 0.472) or response to glucose challenge (Figure 3b; group X timepoint, F_(3,36)_ = 1.15, p = 0.340). These results indicate that sucrose exposure early in life does not alter glucose tolerance when animals are maintained on standard chow in adulthood.

**Figure 4:**
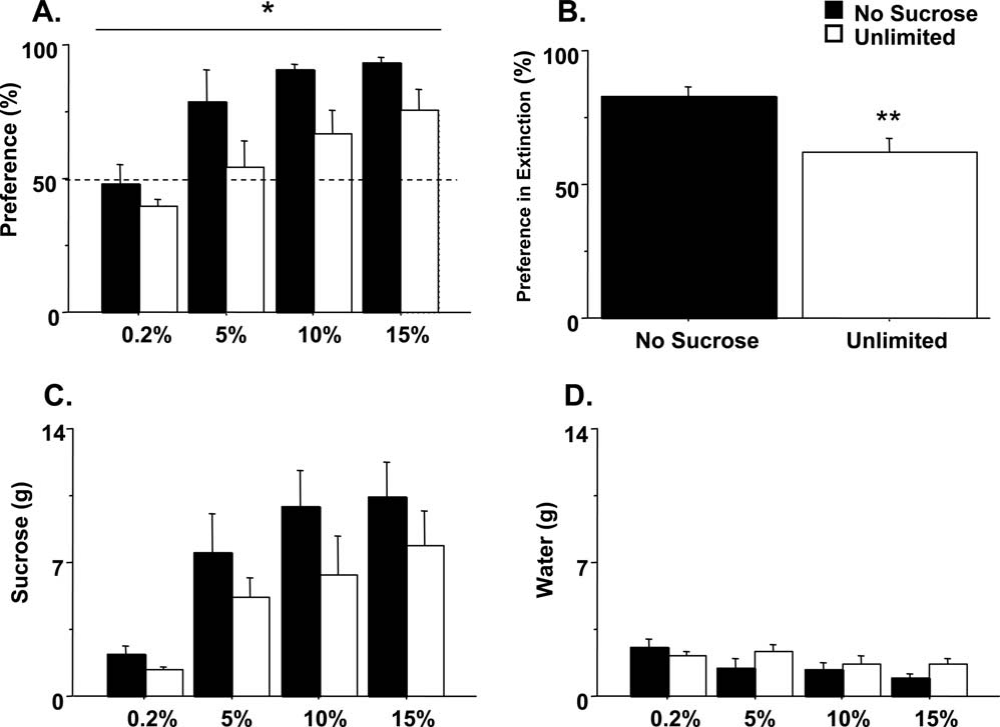
Sucrose preference. (a) Average preference expressed as sucrose consumed (g) divided by total sucrose and water consumption (g) (n = 7). Dashed line indicates no preference. (b) Average preference for the bottle previously paired with sucrose during extinction (n = 7). (c) Average sucrose and (d) water consumption (n = 7) ±SEM; * p<.05, ** p = .005.

Both the progressive ratio and concurrent choice tests evaluate motivation for sucrose when work is required to obtain the reward. We also tested baseline sucrose preference with no explicit work requirement. Singly-housed mice were presented with two bottles, one filled with sucrose and the other with water. Increasing concentrations of sucrose (0.2%, 5%, 10%, and 15%) were presented in one bottle for four days at each concentration. Each day, consumption was measured and the position of the bottles switched to control for position preferences. Across subsequent concentrations, mice exposed to sucrose early in life showed a reduced preference relative to mice raised without sucrose (Figure 4a; Treatment: F_(1,30)_ = 6.0, p = 0.03). However, there were no significant differences in sucrose (Figure 4c; Treatment, F_(1,33)_ = 1.60, p = 0.2) or water (Figure 4d; Treatment, F_(1,36)_ = 1.0, p = 0.3) consumption between groups. Thus, in comparison with the no sucrose exposure group, the mice exposed to unlimited sucrose in early life show mildly reduced sucrose preference resulting in little change in overall consumption. To further assess sucrose-seeking behavior, we tested the mice in extinction conditions. Sucrose bottles were washed, refilled with water, and preference testing was continued for two days. While no overt cues were associated with either bottle, rodents can discriminate bottles based on tactile characteristics [51]. Although both groups showed preference for high doses of sucrose (10 and 15%), mice given unlimited access to sucrose early in life more readily extinguished their preference for the sucrose bottle than animals not exposed to sucrose when they were young (Figure 4b; F_(1,12)_ = 11.54, p = 0.005). Together with the instrumental data described above, these results show a clear reduction in sucrose-seeking behavior in mice given early unlimited exposure. The expression of this effect, however, appears to be contingent upon the costs associated with obtaining the sucrose. In the instrumental tasks where the cost of food is relatively high due to an explicit work requirement and food scarcity, i.e., food-restriction, the effect of sucrose exposure early in life is robust. In contrast, in the sucrose preference test where the work requirement is low and food is freely available, the effect of early sucrose exposure on adult sucrose-seeking is diminished.

### Effects of sucrose exposure on weight gain in adulthood

**Figure 5:**
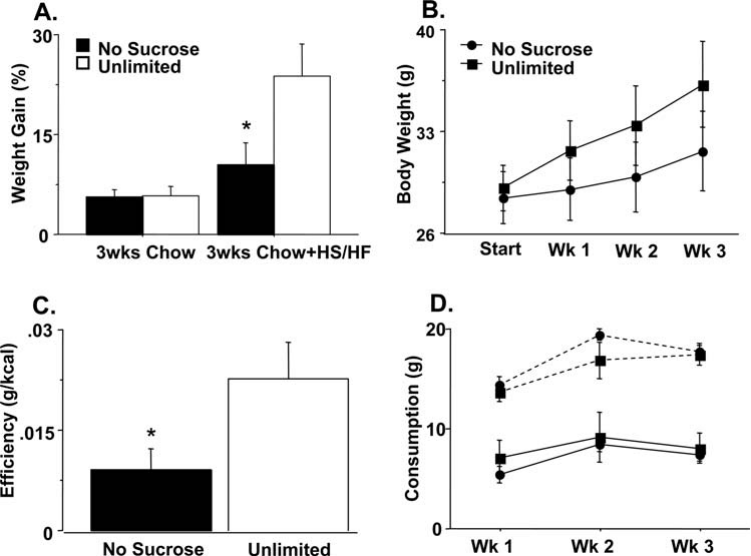
High sugar/high fat dietary challenge. (a) Percent weight gain 3-weeks prior to (left) and during (right) the 3-week HS/HF exposure period in adulthood. (b) Body weight at the beginning and subsequent three weeks of HS/HF dietary options. (c) Metabolic efficiency as gram body weight increase per kcal consumed across the HS/HF dietary challenge. (d) Weekly consumption of standard chow (solid lines) and HS/HF options (dashed lines). N = 5–7; ±SEM, * p<.05.

To directly assess vulnerability to obesity, we measured weight gain when mice had access to freely available high-sugar/high-fat (HS/HF) dietary options. Prior to testing, there was no difference in weight between the groups with no exposure and unlimited exposure to sucrose during early life (F_(1,10)_ = 0.14, p = 0.72). We singly housed the mice and after a one-week acclimation period provided both standard chow as well as HS/HF options consisting of Nestlé® butterscotch, peanut butter, milk and white chocolate chips for three weeks. We found that mice that had unlimited exposure to sucrose when they were young gained more weight in this environment than those animals that did not have access to sucrose during development (Figure 5a; HS/HF weight gain, F_(1,10)_ = 5.84, p = 0.0362; Figure 5b; group X week, F_(3,30)_ = 3.78, p = 0.0206). Though both male and female mice exposed to unlimited sucrose gained more weight than controls in this condition, the effect may be more robust in females (17% and 12% increase over controls in females and males, respectively). While there was no difference between groups in consumption of either the HS/HF food (Figure 5d, dashed lines, F_(1,10)_ = 1.0, p = 0.34) or standard chow (Figure 5d, solid lines, F_(1,10)_ = 0.332, p = 0.577), the sucrose-exposed mice exhibited greater efficiency at storing energy as indicated by weight gained per kcal consumed (Figure 5c, F_(1,10)_ = 5.326, p = 0.0437). Consistent with the sucrose preference test, in an environment where little cost was associated with acquiring the high sugar option, both sucrose exposed and non exposed groups equally preferred the HS/HF diet (as percentage of total consumption, unlimited, 67.1%; no sugar, 71.0%; F_(1,10)_ = 0.551, p = 0.474). Singly housing mice during the dietary challenge is unlikely to have suppressed behavioral differences between the groups as mice were also singly housed during sucrose preference testing where they exhibited behavioral differences.

## Discussion

Our data indicate that a single factor in early, post-weaning development– sucrose exposure– has persistent effects on adult motivated behavior and weight gain. A study in rats published in 1978 [52] found that early exposure to different concentrations of sucrose solution did not alter subsequent adult appetite for sweet foods. Although the authors drew a conclusion opposite ours— that early experience with sucrose does not significantly affect adult consumption and preference— their data are consistent with the present study. Like the Wurtman study, we did not observe an effect of early sucrose exposure on preference for and consumption of sweet food in adulthood when it was freely available. However, their study did not examine whether there was a difference in willingness to work to obtain sweet foods nor did it report the body weights of rats when given access to high-sugar food. Thus, although our study replicates their findings, the more extensive examination of behavior and inclusion of body weight measurement in this study yield a more complex picture of the impact of early experience on adult feeding behavior and body weight and demonstrate that early dietary experience does alter adult consumption and vulnerability to obesity.

Current views on the cause of the recent increase in obesity center on an interaction between genetics and adult environment [5], [19], [24], [48]. The “thrifty genotype” hypothesis proposes that humans are predisposed to store calories in times of plenty in order to survive later times of scarcity. In contemporary society, however, where energy rich foods are readily available without intervening periods of scarcity, this genetic propensity is thought to result in obesity. This view does not consider the effect that dietary experience during development may have on expression of a putative thrifty genotype. In our study, four to seven weeks of exposure to sucrose post-weaning altered adult sucrose-seeking and weight gain among mice that shared an identical “thrifty genotype” and were exposed to identical environments as adults. These data emphasize the importance of a developmental perspective extending beyond gestation and nursing and suggest that children's diets can intensify or ameliorate the impact of a putative thrifty genotype.

In human studies, unlimited soft drink consumption during childhood is correlated with an increase in obesity risk (see [37] for review), consistent with our finding in mice that unlimited access to sucrose early in life resulted in increased weight gain when exposed to freely available HS/HF dietary options. Importantly, however, our study shows that the impact of early sucrose exposure on adult obesity risk depends upon the dietary environment in which the adult finds itself. Sucrose-exposed animals do not seek out sucrose to the extent that naïve animals do when there is a significant cost associated with acquiring it; however, they are more vulnerable to weight gain in an environment with freely-available (low-cost), palatable, energy rich foods. Our study suggests that individuals exposed to sugary diets in early life will be less likely to seek these foods if there are costs associated with obtaining them in adulthood. Consequently, making high sugar and high fat foods less readily available within individual environments, such as the workplace, schools and home, may contribute to effective weight management. These results suggest that with a population raised on a diet high in sugar, environmental manipulation of the costs associated with energy-dense foods is likely to be an efficacious obesity-reducing intervention during adulthood.

These data might suggest that reducing a child's intake of sucrose might diminish future obesity risk. However, human studies have also found that parental restriction of a child's diet also increases obesity risk (see [38] for review). Several potential explanations of these findings have been proposed, all suggesting that increases in sucrose/fat-seeking behavior and consumption follow restriction [38]. The increased motivation to obtain sucrose in the mice not exposed to sucrose in early life― an extreme form of restriction not possible in human studies― supports this notion. The differences in motivation we observe between groups in the behavior tests are not evident when the animals are sated. This, together with the observation of no weight difference while the mice are maintained on standard chow suggest that the motivational differences we observe between the groups reflect alterations in incentive motivation [53], [54] for preferred, sweet food rather than changes in primary motivation, hunger. This is demonstrated clearly in the concurrent choice test where both groups consumed the same amount of total food but the sucrose-exposed mice consumed less sucrose— available, but associated with a cost— showing that the sucrose-exposed mice exhibit less incentive motivation to work for sucrose. This suggests that early experience can alter the incentive motivational processes that determine goal-directed behavior in response to hunger resulting in different behavioral choices and consumption.

The present study cannot determine the mechanisms underlying the observed effects of early sucrose experience. In the behavior tests, differences in sucrose-seeking may arise as a result of (a) different learned valuation of the preferred food arising from early exposure, (b) different metabolic responses to food restriction between sucrose-exposed and non-exposed animals, with the sucrose-exposed mice protecting energy stores to a greater extent or (c) different motivational responsiveness to metabolic signals such as leptin or insulin, which have been shown to interact with midbrain dopamine systems involved in reward-seeking behavior [44], [55], [56]. The glucose challenge data suggests that the two groups respond to acute food deprivation (21 hour fast) and acute glucose increases similarly when maintained on standard chow, consistent with the observation that sucrose-exposure does not effect weight gain when animals eat standard chow. However, differences in energy metabolism and storage may arise in response to chronic food deprivation which may contribute to the observed differences in sucrose-seeking. Similarly, sucrose-exposed and non-exposed mice likely have different metabolic and/or behavioral responses to HS/HF dietary options that promote weight gain, as evidence by their increased feed efficiency. The potential influence of sucrose exposure in early life on the interaction between metabolic signals and motivational systems are currently being investigated.

Although our study indicates the importance of a developmental perspective in studying obesity, with obvious policy implications, many questions remain. If early experience shapes later food-seeking behavior and obesity risk, the question naturally arises as to the time window during which these developmental processes are active and how susceptible the developmental outcomes are to change after that window closes. Establishing and characterizing these developmental phenomena will facilitate investigation into their biological substrates increasing our understanding of how genes and environments interact to produce the current epidemic of obesity.
